# Exercise induced changes in T1, T2 relaxation times and blood flow in the lower extremities in healthy subjects

**DOI:** 10.1186/1532-429X-15-S1-P108

**Published:** 2013-01-30

**Authors:** Juliet Varghese, Debbie Scandling, Chikako Ono, Ashish Aneja, William A Kay, Subha V Raman, Sanjay Rajagopalan, Orlando P Simonetti, Georgeta Mihai

**Affiliations:** 1 Dorothy M. Davis Heart and Lung Research Institute, Wexner Medical Center, The Ohio State University, Columbus, OH, USA; 2 Department of Biomedical Engineering, The Ohio State University, Columbus, OH, USA; 3 Division of Cardiovascular Medicine, Department of Internal Medicine, Wexner Medical Center, The Ohio State University, Columbus, OH, USA; 4 Department of Radiology, Wexner Medical Center, The Ohio State University, Columbus, OH, USA

## Background

Current approaches to evaluate peripheral arterial disease (PAD) overwhelmingly rely on quantification of stenosis severity or flow across stenosis at rest. Although exercise-induced functional alterations in metabolism have been proposed, there is a need to integrate approaches that provide additional risk measures in PAD that can be translated into clinical practice [1]. Recently, quantitative T1/T2 mapping have allowed their usage as risk indicators in the coronary circulation.

To develop analogous risk-markers in PAD, we initially designed experiments to assess the feasibility of an approach combining quantitative magnetic resonance imaging (MRI) relaxometry of skeletal muscle with treadmill exercise testing. We investigated exercise-induced shifts in quantitative skeletal muscle relaxometry measures [2,3] in lower extremity muscle beds in conjunction with quantitative arterial flow to the inflow vessel as an index of conduit vessel flow-reserve in healthy volunteers [4,5].

## Methods

**Figure 1:**
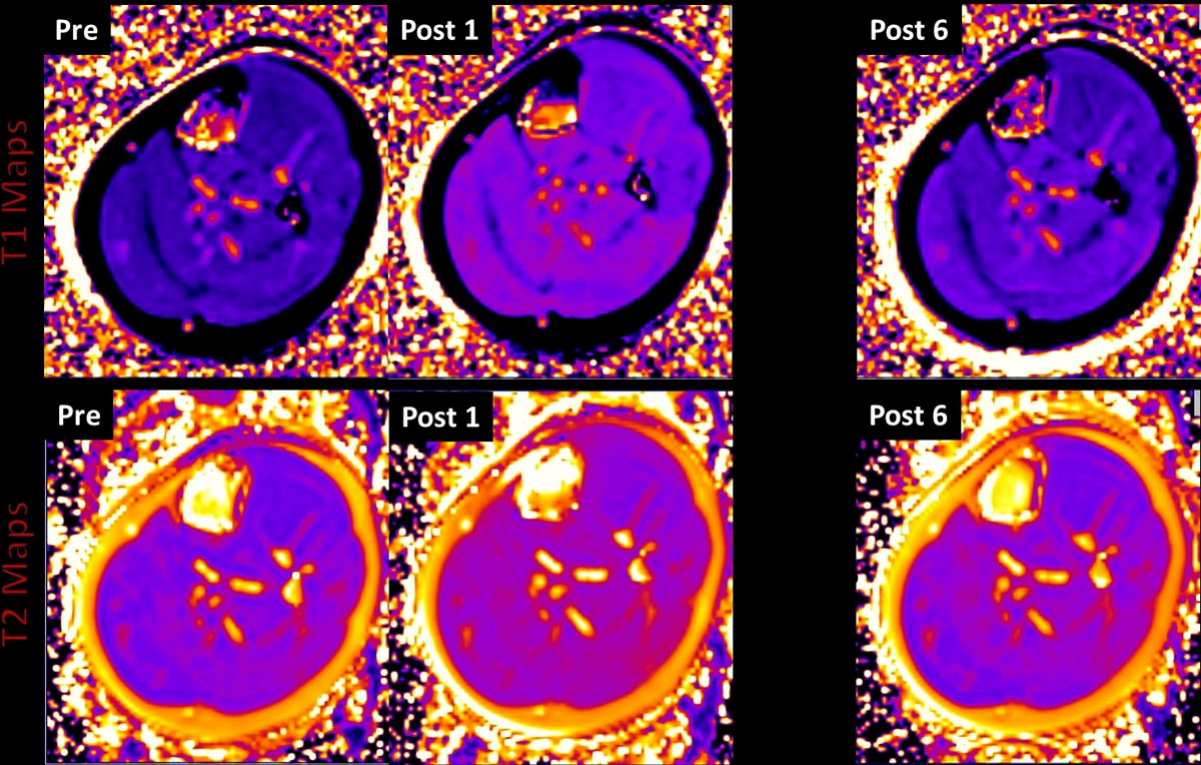
Time course of exercise induced changes in the left calf muscles of one subject. The top row shows T1 maps, while the bottom row shows T2 maps. "Pre" - indicates the baseline map obtained before exercise. "Post 1-6"- indicate the sequence of maps obtained immediately after exercise (~ 4-5 minute intervals).

Fifteen subjects (5 each in age-based groups of 18-30, 30-50 and 50-70 yrs) exercised through Stage 3 Bruce protocol on an MR-compatible treadmill adjacent to the 1.5T MRI system. Axial T1 (TR/TE/TI=1200/1.1/285 ms, FA=60°, 1 NEX, 1.4x1.4x8 mm^3^) and T2 (TR/TEs= 2500/0,24,26,48,60 ms, 3 NEX, 1.4x1.4x8 mm^3^ ) maps of the calf muscles, and blood flow in the superficial femoral artery (SFA) using cardiac triggered 2D cine phase contrast ( TR/TE=35.9/2.7 ms, 2 NEX, 40 heart phases, Venc =120 cm/s, 1x1x6 mm^3^) were obtained at rest, and every ~5 minute intervals for ~30 minutes after exercise. T1 and T2 changes in calf muscles (gastrocnemius, soleus and anterior tibialis) were measured in regions of interest (ROI) on pre and post exercise maps (Figure 1), while blood flow was measured in the SFA supplying this muscle bed. Measured variables (averaged over both legs) were plotted over acquisition time points.

## Results

**Figure 2:**
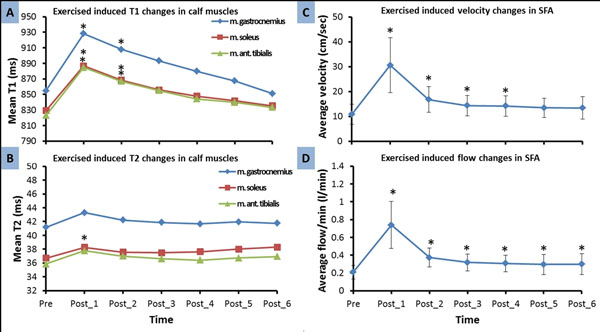
Mean T1 and mean T2 values of the three calf muscle groups (A and B), and superficial femoral arterial (SFA) blood flow (C and D) response to exercise over time, for the entire study population. * p<0.05 - statistical significant difference from pre-exercise

Blood flow and T1 exhibited a significant increase immediately post exercise followed by a progressive decline towards pre-exercise values. T2 only increased significantly in soleus muscle group (Figure 2).

## Conclusions

We have demonstrated the feasibility of assessing exercise induced changes in MRI relaxometric measures and compared these with changes in exercise induced flow in the lower extremities. The changes in T1 and T2 were generally coupled to changes in flow except in older age group some of whom had higher baseline T2 and abnormal post-exercise T2 recovery. We hypothesize that patients at risk for PAD related complications may demonstrate abnormal kinetics of T1 and T2 recovery with exercise due to ischemia induced loss of microvascular integrity and tissue edema.

## Funding

Funding: 10CRP3610010 - American Heart Association

## References

[B1] Kramer C Top Magn Reson Imaging 2007 18 5 357 369 10.1097/rmr.0b013e31815d064c 18025990 PMC2966500

[B2] Yoshioka H Anno I Kuramoto K Magn. Reson. Imaging 1995 13 5 651 659 10.1016/0730-725X(95)00018-C 8569440

[B3] Fleckenstein J Canby R Parkey R AJR 1988 151 231 237 3260716 10.2214/ajr.151.2.231

[B4] Nagaraj H Pednekar A Corros C J. Magn. Reson. Imaging 2008 27 1096 1102 10.1002/jmri.21336 18425829

[B5] Klein W Bartels L Bax L J. Vasc. Surg 2003 38 5 1060 1066 10.1016/S0741-5214(03)00706-7 14603218

